# Proximity-Driven DNA Nanosensors

**DOI:** 10.1149/2754-2726/ace068

**Published:** 2023-07-06

**Authors:** Sara R. Nixon, Imon Kanta Phukan, Brian J. Armijo, Sasha B. Ebrahimi, Devleena Samanta

**Affiliations:** 1 Department of Chemistry, The University of Texas at Austin, Austin, TX 78712, United States of America; 2 Department of Chemistry, Southwestern University, Georgetown, TX 78626, United States of America; 3 Drug Product Development—Steriles, GlaxoSmithKline, Collegeville, PA 19426, United States of America

## Abstract

In proximity-driven sensing, interactions between a probe and an analyte produce a detectable signal by causing a change in distance of two probe components or signaling moieties. By interfacing such systems with DNA-based nanostructures, platforms that are highly sensitive, specific, and programmable can be designed. In this Perspective, we delineate the advantages of using DNA building blocks in proximity-driven nanosensors and provide an overview of recent progress in the field, from sensors that rapidly detect pesticides in food to probes that identify rare cancer cells in blood. We also discuss current challenges and identify key areas that need further development.

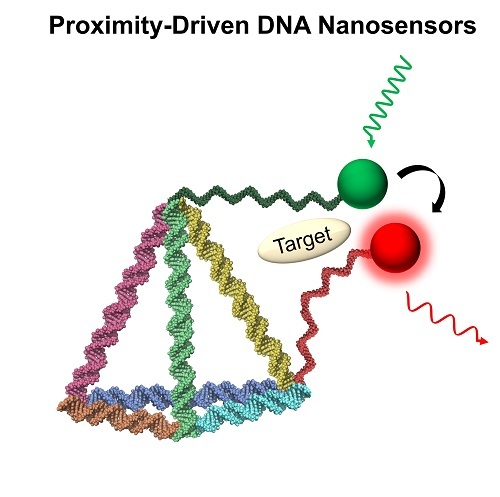

## Highlights

- Proximity-driven DNA nanosensors (PDNs) are widely used in biochemical sensing.
- Ionic strength, temperature, pH, and nucleases can affect the stability of PDNs.
- Targeting moieties can be used to direct PDNs to specific cells and tissues.
- Further research is needed to improve the sensitivity and selectivity of PDNs.
- Further research is needed to translate PDNs into on-site/point-of-care devices.

DNA-based nanostructures—composed either entirely of DNA or of inorganic and organic nanomaterials interfaced with DNA—are transforming chemical and biological sensing.^1^ These materials are highly programmable, modular, biocompatible, and can be synthesized as intricate 3D structures at relatively low costs. Moreover, DNA sequences can be designed to recognize a wide variety of analytes including nucleic acids, ions, proteins, and small molecules. By leveraging these attractive properties, one can tailor DNA-based sensors for detecting targets in samples of varying complexities, ranging from drinking water to whole organisms.^2^ Early work in the field focused on engineering tools for studying analytes in complex media such as human serum. This led to several important breakthroughs, including the development of the Verigene^®^ system, used in hospitals around the world for the rapid diagnosis of infectious diseases.^3^ During the last decade, the focus has expanded to increasingly challenging detection contexts such as the probing of intracellular analytes.^4,5^ These efforts have resulted in platforms that can perform genetic and metabolic analysis of live cells, distinguish cells based on intracellular analyte levels, track mRNA with spatiotemporal resolution, isolate rare cells from whole blood, and detect abnormal scars in vivo.^4,5^

**Figure 1.**
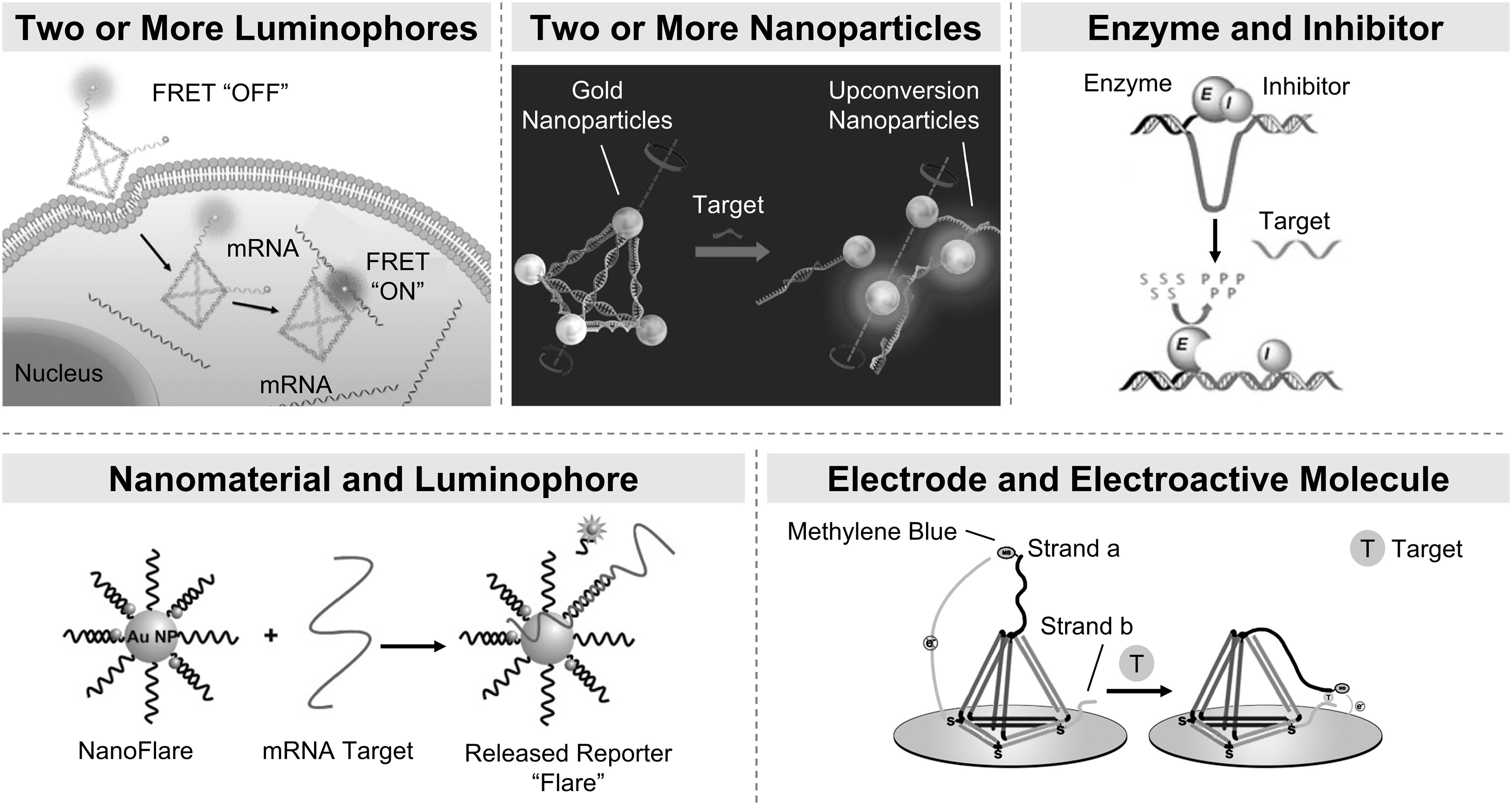
In proximity-driven sensing, interactions between a probe and an analyte produce a detectable signal by causing a change in distance between two or more luminophores (Adapted with permission from Ref. 7. Copyright 2017 American Chemical Society), two or more nanoparticles (Adapted with permission from Ref. 8. Copyright 2016 American Chemical Society), an enzyme and an inhibitor (Adapted with permission from Ref. 10. Copyright 2015 American Chemical Society), a nanomaterial and a luminophore (Adapted with permission from Ref. 9. Copyright 2007 American Chemical Society), or an electrode and an electroactive molecule (Adapted from Ref. 11 with Permission from the Royal Society of Chemistry).

Importantly, the readout interfaced with DNA-based nanostructures determines the sensitivity and selectivity with which target analytes can be detected.^2^ Proximity-driven readouts constitute one such example and are widely used in detection. In proximity-driven sensing, when an analyte interacts with the probe, the distance between two probe components or signaling moieties is altered, producing a detectable change in signal. One of the first DNA-based proximity-driven sensors is the molecular beacon (MB) wherein conformational changes induced by binding to complementary DNA sequences spatially separates a fluorophore and a quencher and results in a fluorescence turn on.^6^ With advances in capabilities, more complex structures have been realized that produce a signal due to a change in distance between two or more luminophores,^7^ two or more nanoparticles,^8^ a nanomaterial and a luminophore,^9^ an enzyme and an inhibitor (or co-factor),^10^ or an electrode and an electroactive molecule^11^ (Fig. 1). The signal observed is typically optical (e.g., a change in color, emission characteristics, or plasmonic coupling) or electrochemical.^2^

In this Perspective, we highlight recent advances in the development of proximity-driven DNA nanosensors (PDNs) with a focus on discussing their advantages and limitations. We also delineate key challenges in this area and outline possible solutions.

## Current Status

Over the last two decades, a large toolbox of DNA-based nanostructures has been developed for sensing. These include constructs made entirely of DNA such as DNA tetrahedrons, tweezers, nanomachines, and diverse 3D structures made with DNA origami.^1^ Probes can also be synthesized by interfacing DNA with other inorganic or organic nanomaterials with the goal of exploiting their beneficial optical, electrical, magnetic, or catalytic properties.^2^ Such probes have been used for chemical and biological sensing across a diverse set of samples including environmental specimens, food products, soil, and complex biological milieu.^2^

The DNA in these structures can play multiple roles, including as a recognition element for sensing the chemical species of interest,^9,12^ as a molecular ruler for positioning signaling moieties with sub-nanometric precision,^13^ or as a trigger for signal transduction.^14^ Moreover, DNA can promote biological interactions that enable the probe to access its target. For example, nanoparticles densely functionalized with DNA are readily taken up into cells through receptor-mediated endocytosis—a phenomenon that has been leveraged for developing intracellular probes.^15^ DNA sequences such as aptamers can themselves act as targeting moieties which can be utilized to drive preferential accumulation of probes at sites of interest.^13^ Importantly, probes incorporating targeting moieties can not only be directed to specific tissues and cells, but also programmed to enter cells along distinct pathways and reach different sub-cellular organelles.^13,16^

The synergy of DNA-based nanostructures and proximity-driven sensing has given rise to PDNs with new and unprecedented capabilities. In one regard, these structures have driven important technological advances. For instance, the NanoFlare constituted the first platform capable of measuring the mRNA content of live cells at single-cell resolution.^9^ These structures consist of a gold nanoparticle that acts as a quencher and is densely functionalized with recognition sequences for a specific target, hybridized to short, fluorophore-labeled “flare” sequences. Target binding to the recognition strands displaces the flare sequence, separating the fluorophore and gold nanoparticle, and turns on fluorescence. NanoFlares represented a particularly important advance as they could enter cells without transfection reagents and have since been used in several clinical applications, including as probes capable of detecting and isolating circulating tumor cells in live form based on genetic markers.^17^ In another regard, PDNs have enabled fundamental discoveries in the biological sciences. A key example is represented by the use of DNA nanomachines to identify the first calcium ion importer in the lysosome of animals.^18^ Significantly, DNA nanomachines can package a targeting moiety, sensing module, and normalizing agent into a single structure to allow for absolute quantification of analytes in specific organelles.^13,16^ Other studies have further shown that aptamer-based electrochemical PDNs can be used for continuous monitoring of biologically relevant molecules in whole blood and living organisms (e.g., drug concentrations in blood and interstitial fluid).^11,19^ Notably, nanostructured probes can overcome background signal often observed with electrochemical sensing.^11^

Thus far, the field has focused largely on the design of new probe structures and establishing proof-of-concept that these materials are suitable for targets in various media with progressively increasing complexity. In more recent years, the field has gradually moved toward achieving two broad goals: (i) realizing probes for on-site analyte detection (e.g., for point-of-care (POC) diagnostics or detection of pesticides in agricultural products)^20–22^ and (ii) developing structures that enable quantitative sensing in living cells across multiple length scales (i.e., from sub-cellular to whole organism).^4,5^

## Future Needs

PDNs may be used to (i) simply report the presence of an analyte in a sample (e.g., a COVID test), (ii) compare the levels of an analyte in two different specimens (e.g., RNA expression levels in two cell lines), (iii) quantitate the absolute amount of an analyte (e.g., the amount of mercury in lake water), or (iv) track an analyte with spatiotemporal resolution (e.g., the localization of chloride ions in lysosomes). These sensing contexts coupled to the environment in which the detection must occur (e.g., drinking water vs inside a living cell) drives the probe design and determines its stability needs. It also informs the considerations necessary to maximize sensitivity and selectivity while promoting speed and accessibility of the assay (Table I). We discuss these aspects in greater detail below and highlight future needs.

**Table I.** Factors that impact PDN attributes.

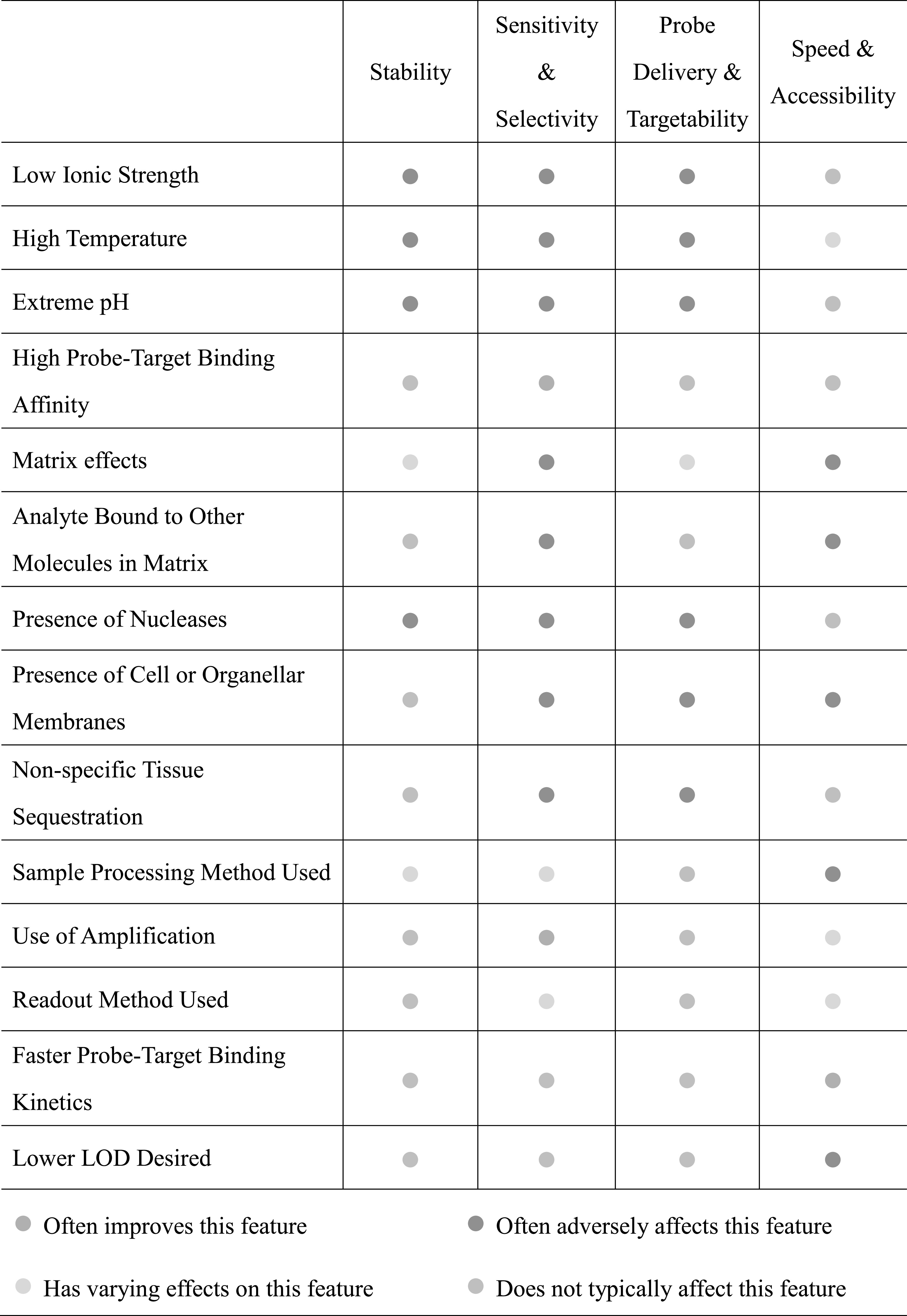

## Improve the Stability of PDNs

Several factors such as ionic strength, temperature, pH, as well as the detection medium can affect the stability of PDNs. One of the most fundamental solution properties is ionic strength as it impacts the folding of DNA and its binding to complementary sequences. Specifically, cations are necessary for DNA hybridization as they can interact with the DNA backbone and screen the negative charge of the phosphate groups. This is especially important for PDNs that utilize DNA origami or supramolecular DNA structures as they require the precise assembly of multiple DNA strands.^23,24^ For in vitro samples (e.g., lake water, soil, cell lysates, and biofluids), the salt concentration can be adjusted by the addition of appropriate buffers. However, special considerations must be made in vivo (e.g., live cells and tissues). Many DNA-based nanostructures require relatively high concentrations of divalent cations such as Mg^2+^ to form and therefore these structures are prone to disassembly under physiological conditions.^24^ Consequently, an area that requires future work is the design of PDNs that are resistant to low-salt concentrations.

In this regard, certain wireframe DNA nanostructures have been shown to maintain stability under reduced salt concentrations.^25^ Concatenation to form superstructures is another potential avenue toward mitigating this challenge.^26^ Recently, several reports have shown that DNA strands can be crosslinked by incorporating light-sensitive groups or by using chemical crosslinkers.^27,28^ The resulting structures show stability in a wide variety of aqueous and non-aqueous solvents. Such structures with improved stability could potentially be used for long-term sensing.

Similarly, pH is also important for the stability of PDNs. While DNA-based nanostructures are typically stable over a pH range of ∼5 to 11,^23,29^ variation in pH could potentially result in their degradation (e.g., hydrolysis or depurination of DNA), changes in structure (e.g., if the DNA sequences contain C-rich structures capable of forming i-motifs) or shifts in binding affinity to targets. These considerations are especially important for intracellular sensing, as the large majority of PDNs are taken into cells via endocytosis and can encounter pHs as low as 4 in lysosomal compartments.

Moreover, PDNs are sensitive to thermal changes owing to the fact that DNA folding and hybridization are temperature-dependent. This means that the optimal conditions for maximizing signal-to-noise ratios will vary between different sequences and undesired temperature-induced structural changes or dehybridization can lead to erroneous readout. Temperature is particularly important when validating structures in buffer for later use *in cellulo*. Specifically, probe performance should be evaluated at ∼37°C, rather than at room temperature, to more closely mimic conditions in cellular contexts.

Finally, the degradation of PDNs, especially in biological samples containing nucleases, is a cause for concern. While DNA-based nanostructures are less susceptible to degradation compared to linear DNA sequences, their stability can be further enhanced by modifying the nucleotide base (e.g., the use of unnatural nucleotide bases), the sugar ring (e.g., 2′-O-methyl), or the phosphate backbone (e.g., phosphorothioate).^4^ Nuclease degradation can also be attenuated through modifications to the supramolecular structure, (e.g., incorporating multiple crossover motifs),^30^ encapsulation into lipid bilayers^31^ or DNA-based cages,^32^ and functionalization with polymer coatings.^33^

## Enhance Sensitivity

The sensitivity with which a probe can detect an analyte depends on several factors, such as the binding affinity between the probe and the analyte, the readout technique used, as well as interference from other molecules in the sample. A stronger binding affinity promotes interactions between the probe and the analyte even at low concentrations, thereby improving the limit of detection (LOD).

The readout strategy also determines sensitivity. For example, colorimetric methods typically enable target detection in the micromolar to millimolar range, although the incorporation of nanoparticles with large extinction coefficients (e.g., gold nanoparticles) has been shown to push LODs down to nanomolar concentrations.^34,35^ On the other hand, techniques such as fluorescence and surface-enhanced Raman scattering (SERS) are capable of single-molecule sensing, and as such are routinely used for detecting analytes in the attomolar to femtomolar range.^36–38^

In complex media, matrix effects can significantly reduce signal-to-noise ratios due to non-specific interactions of the probe or analyte with other components in solution. To overcome matrix effects, masking agents or extraction steps could be used. Certain targets such as nucleic acids can be subjected to pre-amplification through polymerase chain reaction (PCR) or rolling circle amplification (RCA) to increase their concentrations prior to detection. Alternatively, readouts that are less prone to matrix effects, such as electrochemiluminescence, can be used.^39,40^ However, these strategies are often not suitable for every detection setting. For example, electrochemical methods necessitate the use of electrodes which are inconvenient from the standpoint of speed, throughput, and invasiveness for long-term intracellular or deep-tissue sensing. To enhance the LOD, the signal generated can be amplified by incorporating mechanisms such as hybridization chain reaction^41^ or by using enzymatic reactions that are triggered by target-probe interactions.^14^

## Enhance Selectivity and Reduce False Positive Signal

The probe and the analyte must be accessible to each other for sensing to occur—otherwise false-positive or false-negative signal is possible. Therefore, for targets that are complexed to other biomolecules (e.g., divalent metal ions that are often strongly bound to proteins with ∼ nanomolar dissociation constants), an extraction step is necessary before they can be detected and quantitated. Similarly, unintended binding of probes to other chemical species (e.g., structurally similar molecules) can make it prohibitively challenging to detect the analyte of interest. In addition, matrix-induced changes in the probe structure (e.g., due to variation in ionic strength or nuclease degradation) can change the proximity of the signaling moieties even in the absence of the target. This issue is particularly relevant in biological matrices where nuclease digestion can lead to false positives, especially in fluorophore/quencher-based detection systems.^15,42^ False positives can also occur during intracellular sensing due to cellular autofluorescence,^4,5^ organellar accumulation of dyes, or electrostatic interaction with organellar membranes.^43^

These difficulties can be mitigated in several ways. For example, agents that disrupt interactions with interfering molecules could be added to minimize false-positive/negative signals. Alternatively, recognition sequences that bind to targets with both higher affinity and specificity could be used to promote preferential binding to the target analyte in comparison to interfering species. The use of red-shifted dyes can overcome cellular and tissue autofluorescence. However, in situations where unintended changes in the distance between sensing moieties is unavoidable, PDNs may not be the right choice. In this respect, forced intercalation (FIT) has shown great potential.^15,44^

## Improve Probe Delivery and Targetability

For biological sensing, barriers may be present which preclude the interaction of the probe and the target. For example, in the context of intracellular sensing, the cell membrane poses a physical barrier to probe delivery as linear DNA sequences do not readily enter cells without transfection reagents.^4,5^ Certain DNA-based nanostructures, however, are internalized in high quantities through receptor-mediated endocytosis,^9^ and therefore could be used as intracellular probes. Spherical nucleic acids constitute one such example wherein the dense DNA functionalization around a nanoparticle core enables cellular uptake and subsequent detection and tracking of various biomolecules such as mRNA, ATP, and glucose.^4,5,9,15^

Once within cells, accessing analytes in different cellular compartments presents an additional hurdle, which could be ameliorated via the inclusion of targeting moieties (e.g. aptamers, small molecules, peptides, etc.) into PDNs.^13,16,45^ In this regard, DNA nanomachines labeled with MUC1 aptamer and cholesterol have been shown to allow for quantitative imaging of nitric oxide in the trans-Golgi network and plasma membrane, respectively.^13^

For in vivo applications, probe delivery and target accessibility are especially difficult, as DNA-based nanostructures are prone to opsonization and sequestration in organs such as the kidney and liver.^46^ As before, targeting moieties can assist in controlling their localization.^46,47^ For example, DNA-based nanostructures with the transferrin aptamer have been shown to cross the blood brain barrier.^48^ However, examples of such structures for in vivo detection and imaging are scarce.^47^

## Increase Speed and Accessibility

The speed and accessibility desired of PDNs is dictated primarily by the detection setting (e.g., centralized facilities, in the field, on-site, or at the POC) and sample type (e.g., cells, lake water, agricultural products, or serum). It should be noted that speed and sensitivity are often competitors, as more rapid readouts tend to have lower sensitivity (e.g., colorimetry). However, in many scenarios including on-site/POC testing, speed is a key consideration.

Detection platforms that require sample treatment, such as a purification, extraction, or incubation step before analysis will naturally provide slower readouts. Furthermore, probes that are kinetically slow in transducing signal following analyte recognition can be challenging to use in on-site/POC situations. This is also important to weigh in cellular contexts, where a probe must be able to yield signal with sufficient speed to enable studying analytes that dynamically change in concentration or spatial positioning.^4^

Accessibility is also an attribute that should be carefully considered during the development of detection platforms, especially for on-site/POC use. Therefore, a key area of research is in the development of probes that yield appropriate LODs while obviating the need for complex equipment.^49^ Strategies based on colorimetric readouts are particularly well-suited for this, as signaling can be achieved using simple instrumentation (e.g., a plate-reader) or no instrumentation at all (e.g., naked-eye detection with a lateral-flow assay). However, one challenge lies in the analysis of complex samples (e.g., serum), where the natural color of the sample may make it difficult to see the evolution of color triggered by an analyte recognition event. The use of electrochemistry, fluorescence, and certain plasmonic-based methods can also afford platforms capable of interfacing with portable, handheld devices.

## Conclusions

PDNs have allowed for sensing a wide variety of biomolecules encompassing ions, small molecules, proteins, and nucleic acids in complex samples. However, several challenges must be addressed in order to unlock their full potential. Strategies are needed to further enhance the stability of PDNs under various solution conditions, such as low-ionic strength environments or nuclease-containing media. Designing probes that are highly sensitive and selective but do not require multiple processing steps or complex instrumentation is another hurdle. PDNs promoting speed and accessibility are especially important for the development of POC diagnostics. Additional considerations are necessary for in vivo detection where long-term probe stability, ability of the PDNs to reach their target sites, and background signal from tissues are relevant concerns. We envision that solving these challenges and further developing new classes of PDNs that sense multiple targets simultaneously, package sensing and therapeutic modalities into one structure, and enable the continuous monitoring of targets will result in a paradigm shift in biology and medicine.
